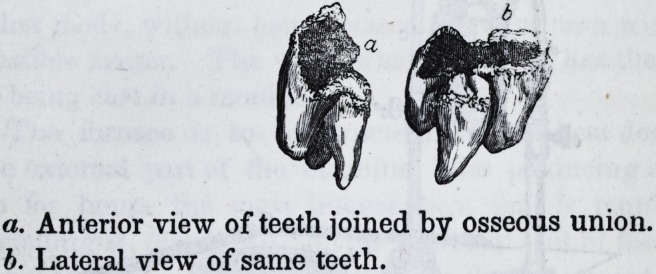# Osseous Union of Teeth

**Published:** 1845-03

**Authors:** James D. McCabe

**Affiliations:** Richmond, Va.


### ARTICLE VI.

*Osseous Union of Teeth.* By James D. McCabe, D. D. S., of Richmond, Va.

The connection of the teeth, two or more, by a true bony union of their fangs or sides, though of no practical importance, has, nevertheless, derived some consideration from the fact, that Dr. Koecker, a writer of no small notoriety in the profession, has endeavored to throw discredit upon its existence, in declaring that he had never in "*all*" *his* "*practice* for many years," "been able to obtain occular demonstration of such a fact, or to satisfy himself that there ever had been such a case," and asserting, as the deliberate conviction of his judgment, that "there is no other way of accounting for such doctrine, than by attributing it to a weak credulity, or love of the marvellous, or a desire to impose upon the world." There are, however, "many things in nature, not dreamed of in" the philosophy of Dr. Koecker, and the unimpeachable and undoubted testimony of a number of *scientific* practitioners, describes to us, numerous cases of that peculiar kind, which the doctor, in "all" his "practice for many years," had never been able to obtain occular demonstration of.

*a.* Anterior view of teeth joined by osseous union.
*b.* Lateral view of same teeth.

The above drawing presents a most distinct and beautiful case of osseous union between the second and third molaris. The teeth were extracted by Dr. Robert Early, an accomplished dentist, of Lynchburg, and the drawing was made by a young artist of that town, and kindly presented to me by Dr. Early. The union is most perfect along the whole extent of the root, and portion of the crown, and presents the same uniform appearance from the apex of the fang to the crown of the tooth, that the other parts of the tooth-bone exhibits; indeed, it is the most perfect specimen of the osseous union I have ever seen. If there were no other cases furnished by the writings of the profession, this alone would be sufficient to establish most conclusively, the existence of such a condition of the teeth. If this is worth a place in your journal, give it a location there.

## Figures and Tables

**Figure f1:**